# The Diagnosis of Intestinal Fibrosis in Crohn’s Disease—Present and Future

**DOI:** 10.3390/ijms25136935

**Published:** 2024-06-25

**Authors:** Sara Jarmakiewicz-Czaja, Jolanta Gruszecka, Rafał Filip

**Affiliations:** 1 Institute of Health Sciences, Medical College of Rzeszow University, 35-959 Rzeszow, Poland; sjczaja@ur.edu.pl (S.J.-C.); jagrusz@onet.pl (J.G.); 2 Department of Clinical Microbiology, Clinical Hospital No. 2, 35-301 Rzeszow, Poland; 3 Institute of Medicine, Medical College of Rzeszow University, 35-959 Rzeszow, Poland; 4 Department of Gastroenterology with IBD Unit, Clinical Hospital No. 2, 35-301 Rzeszow, Poland

**Keywords:** Crohn’s disease, diagnosis, intestinal fibrosis, miRNAs, molecular testing

## Abstract

Crohn’s disease (CD) progresses with periods of remission and exacerbations. During exacerbations, chronic inflammation leads to tissue destruction. As a result, intestinal fibrosis may develop in response to the ongoing inflammatory process. Fibrosis in CD should be considered the result of the response of the intestinal wall (over) to the presence of inflammation in the deep structures of the intestinal wall. In the absence of ideal noninvasive methods, endoscopic evaluation in combination with biopsy, histopathological analysis, stool analysis, and blood analysis remains the gold standard for assessing both inflammation and fibrosis in CD. On the contrary, the ability to identify markers of intestinal fibrosis would help to develop new diagnostic and therapeutic methods to detect early stages of fibrosis. It is speculated that miRNAs may, in the future, become biomarkers for early noninvasive diagnosis in the treatment of intestinal fibrosis. The purpose of this review is to summarise existing diagnostic methods for Crohn’s disease and present recent scientific reports on molecular testing.

## 1. Introduction

Crohn’s disease (CD) is included in the group of inflammatory bowel diseases. It occurs during periods of exacerbation and remission of the disease. It is a chronic disease entity, and the granulomatous form is often present (granulomas in the lamina propria are composed of macrophages and monocytes). The incidence of granulomas in CD patients is associated with a more frequent location in the ileum, colon, and upper gastrointestinal tract. Furthermore, the need for biologic therapy was significantly more frequent in patients with granulomas [1,2,3]. Inflammation can affect any part of the gastrointestinal tract, from the mouth through the oesophagus, stomach, small intestine, large intestine, and rectum. Inflammation in CD is fragmented, which means that lesions are separated by healthy fragments. Inflammation is most often located in the terminal segment of the small intestine and the initial colon. In the early stages, the disease process involves the intestinal mucosa, followed by subsequent layers of the gastrointestinal wall (muscle fibres, peritoneum) [4,5,6,7]. Increasingly, researchers are turning their attention to the process of gastrointestinal fibrosis in CD. The process is associated with the deposition of extracellular matrix (ECM). An increase in the number of myofibroblast cells in conjunction with smooth muscle proliferation can lead to intestinal strictures and obstructions [8]. Crohn’s disease can occur in both men and women of any age, diagnosed primarily between the ages of 20 and 30 [9]. The incidence of CD, compared to the incidence of ulcerative colitis (UC), is similar [10]. Currently, 20–30 cases per 100,000 people are diagnosed in Western countries. The epidemiology of CD incidence can vary by age [11].

The clinical assessment of disease activity is based on several tools, one of which is the Crohn’s Disease Activity Index (CDAI). It is considered the gold standard for assessing CD activity. The activity of the disease, according to CDAI, is divided into mild, moderate, and severe. The assessment includes the severity of abdominal pain, the percentage of weight loss, and patient well-being, among other factors. Disease remission using CDAI is determined when the index is below 150 points, among other things [12]. Table 1 shows the CDAI scores for the clinical evaluation of disease activity.

Another tool to assess CD activity is the Montreal classification. It is a modification of the Vienna classification [14]. Table 2 shows the Montreal classification.

Patients experience general symptoms such as abdominal pain, fever, weight loss, diarrhoea, and, sometimes, rectal bleeding [18,19]. Most often, inflammation is localised in the small intestine with the involvement of the terminal ileum [20]. One-fifth of patients with CD may develop fistulas or strictures of the bowel [21]. Depending on the location of inflammation, CD can also manifest itself in the oral cavity (gingivitis or inflammation of the corners of the lips) and in the upper gastrointestinal tract, for example, esophagitis, dysphagia, odynophagia, gastritis, dyspeptic symptoms [22,23]. CD can also have extraintestinal manifestations, such as hepatobiliary, ocular, cutaneous, and musculoskeletal manifestations [24,25].

## 2. Aetiology of Crohn’s Disease

Despite great progress in research on the aetiology of CD, it is not yet fully elucidated. Several studies indicate significant links between environmental factors that influence the onset of intestinal dysbiosis and genetic and immunological factors, among others.

### 2.1. Immunological Factors

Immunopathogenesis in CD is closely related to mutations in certain genes responsible for the defence of the body against pathogens and environmental factors. One of the predisposing factors to the onset of CD is an inadequate response of the immune system to micro-organisms of the intestinal microbiota. The innate immune response, which includes dendritic cells (DC) and macrophages, is the first line of defence against pathogens entering the human body. DCs, with the help of Toll-like receptors (TLR), after contact with pathogen-associated molecular patterns (PAMP), present an antigen that is responsible for activating T lymphocytes, thus inducing an immune response [26]. The direct barrier to micro-organisms is the mucosal layer that covers the intestinal epithelium. It prevents the penetration of bacteria. Disruption of mucosal barrier function has been observed in CD patients through reduced mucin secretion due to reduced expression of MUC mucin antigens 3, 4, and 5 [27]. CD4+ T cells can induce inflammation and maintain inflammation in the mucosa by producing pro-inflammatory cytokines IL-12, IL-21, and IL-23, playing an important role in CD [28]. The dysregulation of the immune response is closely related to genetic factors that predispose to CD [29].

### 2.2. Genetic Factors

Genome-wide association studies (GWASs) have made it possible to locate single-nucleotide polymorphisms (SNPs), thus detecting components that are responsible for the genetic predisposition of the occurring diseases. In the early 2000s, the first susceptibility to CD was linked to a polymorphism of the nucleotide binding oligomerization domain containing 2 (*NOD2*) gene. The *NOD2* gene encodes a protein that functions as a receptor that recognises the building blocks of pathogenic bacteria. It is found in intestinal epithelial cells, Paneth cells, and monocytes, where it stimulates their autophagy. When the gene is mutated, autophagocytosis induction is impaired, which can lead to the impaired recognition of harmful pathogens in the body and their spread [30]. Other autophagy gene mutations (autophagy-related 16-like 1 (*ATG16L1*) and immunity-related GTPase family M protein (*IRGM*) that can predispose to inflammatory bowel disease (IBD) are also presented in the literature [31]. Another genetic risk factor for CD is mutations in *PTPN22* (Protein tyrosine phosphatase non-receptor type 22) and *CLEC7A* (C-Type Lectin Domain Containing 7A) mutations [32,33]. The rates of CD inheritance concordance in monozygotic twins are about 50% [34]. In addition to genetic factors, in recent years, researchers have also pointed to the importance of linking epigenetic factors to the predisposition to develop CD, that is, deoxyribonucleic acid (DNA) methylation, post-translational modifications of histone proteins, and expression of noncoding ribonucleic acid (RNAs) [35,36,37]. They are usually triggered by different types of environmental factors, again pointing to the links between the various components that can cause CD [38].

### 2.3. Environmental Factors Influencing the Occurrence of Intestinal Dysbiosis

Another component that can predispose one to CD is intestinal dysbiosis, which is a quantitative and qualitative disorder in the intestinal microbiota.

#### 2.3.1. Diet

Many factors affect the composition of the intestinal microbiota, one of which is diet. The composition of the diet can induce changes in the composition of the intestinal microbiota, both in the short term and long term [39]. One of the most studied food components is dietary fibre. A diet high in fibre can increase the number of commensal bacteria (e.g., *Faecalibacterium* sp., *Lactobacillus* sp., *Akkermansia* sp.) while decreasing the number of pathogenic bacteria (e.g., *Enterobacteriaceae*). Furthermore, the breakdown of dietary fibre by intestinal micro-organisms results in the production of beneficial SCFAs (short-chain fatty acids). These are mainly composed of butyrate, propionate, and acetate. Butyrate has many health benefits; among other things, it is a major source of energy for intestinal epithelial cells, and it strengthens the function of the intestinal barrier and, thus, can exert an immunomodulatory effect [40,41]. On the contrary, a Western-type diet loaded with significant amounts of fat, especially saturated or trans fatty acids, can cause changes in intestinal microbiota composition, increase intestinal permeability, and predispose one to colitis [42,43,44]. In turn, high omega-3 intake can indirectly increase the amount of *Lachnospiraceae* and *Ruminococcacae*, which are involved in SCFA production [45,46]. Also important is a group of polyphenols that show antioxidant and anti-inflammatory activity. Some of the substances show the ability to inhibit the proliferation of pathogenic bacteria while promoting the growth of commensal bacteria [47,48,49]. The Mediterranean diet is considered one of the best diets in the world. The main principles of this diet are the consumption of whole grain products and large amounts of vegetables, fruits, fish, seafood, and olive oil while reducing the amount of meat consumed, especially red meat; alcohol; or highly processed foods. The American Gastroenterological Association (AGA) recommends a Mediterranean diet for patients with IBD, unless there are contraindications to dietary modification [50].

#### 2.3.2. Drugs

Another factor is the medications taken. These can have bacteriostatic and bactericidal effects. Examples of such drugs that alter the composition of the intestinal microbiota are antibiotics, laxatives, antidepressants, statins, and proton pump inhibitors [51]. However, the interaction between the gut microbiota and the drugs used is bidirectional. Micro-organisms can affect the pharmacomicrobiomics of a drug, that is, its bioavailability or toxicity [52]. Interactions of the intestinal microbiota with drugs can be indirect, for example, competition for the metabolism of enzymes, and direct, for example, biochemical transformation of drugs [53]. Zimmermann et al. point out that it would be necessary to identify the gene products of gut microbes that metabolise drugs in order to administer the appropriate pharmacotherapy to an individual [54].

#### 2.3.3. Physical Activity

An additional factor may be the level of physical activity. Campaniello et al., in their article, indicate that physical activity can change the gut microbiota qualitatively and quantitatively. The composition of the microbiota depends not only on diet and environment but also on the intensity of physical activity and the type of exercise performed [43,55]. Marttinen et al. indicate that physically active people have a higher amount of *Prevotella, Akkermansia*, and *Veillonella* bacteria [56]. However, micro-organisms that reside in the intestinal tract can also have an impact on human performance [57,58]. Ramos et al. indicate that the introduction of exercise in the elderly has the potential to alter the beneficial gut microbiota but also note that further research is needed in this area [59]. Leading an active lifestyle has the effect of increasing the diversity of intestinal micro-organisms, including beneficial types of bacteria, in addition to improving the functioning of the intestinal barrier and mucosal immunity [60]. Furthermore, the gut microbiota can indirectly modulate motivation to perform physical activity [61].

#### 2.3.4. Stress

In addition, it has been shown that when exposed to chronic stress, intestinal microbes are involved in physiological consequences, that is, dysregulation of the HPA (hypothalamus–pituitary–adrenal axis) and disruption of normal intestinal barrier function [46]. Dumitrescu et al. indicate that intestinal dysbiosis can induce oxidative stress, which, in turn, can exacerbate dysbiosis [62]. Humbel et al. showed that feelings of stress and anxiety are associated with a lower diversity of the gut microbiota [63]. People with lower levels of daily stress show a lower risk of IBD. Moreover, in addition to mental tension, depression or anxiety can worsen the course of the disease [64]. The number of exacerbations of the disease has also been shown to be related to deteriorating mental health [65].

#### 2.3.5. Sleep Disorders

In addition, sleep disorders are a factor that causes changes in the gut microbiota. Wang et al., in their article, present that the microbiota–gut–brain axis plays a significant role, both directly and indirectly, in the pathogenesis of sleep disorders [66]. In another paper, the authors indicate that intestinal dysbiosis may contribute to cognitive deficits caused by sleep disorders [67]. Furthermore, fragmentation of sleep can disrupt the proper functioning of the intestinal barrier [68]. Patients with CD are more likely to have poor sleep quality compared to patients with UC. However, in both groups, sleep disturbances occur even in patients in remission of the disease, leading to a deterioration in their quality of life [69]. Chronic sleep disorders are often associated with depression, particularly in women [70]. Some researchers suggest that the manipulation of the gut microbiota may be a potential factor in improving sleep quality [71,72].

#### 2.3.6. Smoking

Another component that disrupts the commensal microbiota is smoking tobacco products, but this is a two-way effect. Potential mechanisms are metabolic biomarkers that are related to neurotransmitters and the positive feedback of smoking [73]. Furthermore, tobacco smoke can alter the normal function of the intestinal barrier [74]. Intestinal dysbiosis caused by smoking can predispose one to several diseases. However, as the authors point out, the effects of a particular type of cigarette (electrochrome/traditional) and smoking conditions (passive/active smoker) on the gut microbiota should also be investigated [75].

**Figure 1:**
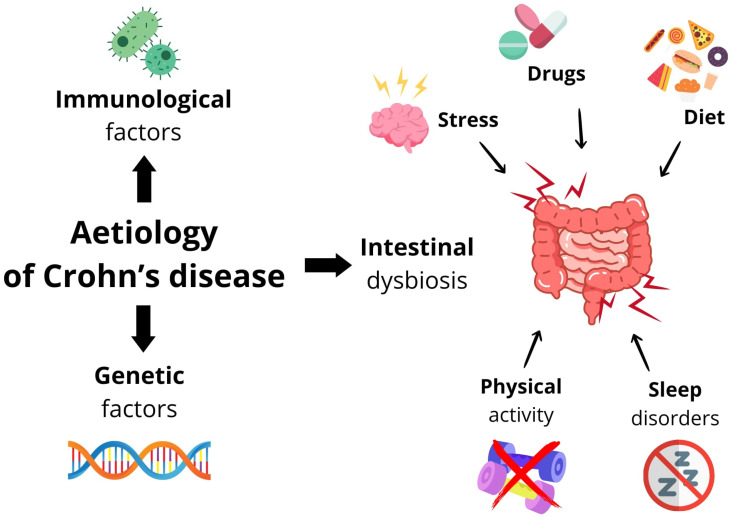
Summary of aetiological factors in Crohn’s disease.

A summary of the aetiological factors of Crohn’s disease is shown in Figure 1.

## 3. Diagnostics

Chronic inflammation in CD causes a disruption of the epithelial barrier and tissue destruction. Fibrosis, which is a healing mechanism, becomes progressive and deleterious in long-term disease, in which persistent tissue damage and healing result in scar tissue [76]. At the tissue and cellular level, fibrosis is the result of an abnormally enhanced response to chronic damage to the intestinal wall, characterised by the excessive accumulation of collagen-rich extracellular matrix (ECM) produced by the increase in the number of mesenchymal cells. Fibrosis is common in the natural history of IBD and accounts for most complications, such as strictures, intestinal penetration, and obstruction, which often require surgery [77]. In the advanced stage of the disease, fibrosis occurs, leading to segmental narrowing of the intestinal lumen. Above the strictures, lumen dilatation and wall hypertrophy appear [78,79,80,81,82].

In the absence of ideal non-invasive methods, endoscopic evaluation in combination with biopsy, histopathological analysis, stool analysis, and blood analysis remains the gold standard for assessing inflammation and fibrosis in CD [83,84].

### 3.1. Endoscopic and Histopathological Examinations

Endoscopic and histopathological examinations may involve the following:

– Upper gastrointestinal tract (gastroscopy);
– Lower gastrointestinal tract (colonoscopy and ileocolonoscopy);
– Small intestine (enteroscopy and endoscopic capsule).

During examinations, mucosa fragments are taken for histopathological examination, allowing the type and activity of inflammation to be evaluated. Endoscopic and histopathological examination of the collected sections allows for not only a correct diagnosis but also a more precise assessment of disease progression, evaluation of the effectiveness of treatment, and diagnosis of precancerous and neoplastic lesions [79,80,81,83,85].

### 3.2. Imaging Tests

Fibrosis in CD patients can cover the entire thickness of the intestinal wall, including the mucosa, submucosa, intestinal muscle, and the serous membrane layer. Intestinal fibrosis is one of the leading causes of failure in treatment and hospitalisation for surgical resection. The goal of many studies is to change intestinal fibrosis from a static and irreversible state to a dynamic and reversible disease. Therefore, promising imaging technologies or biomarkers for the detection of intestinal fibrosis to initiate new antifibrotic drugs and even predict the onset of intestinal fibrosis [86,87,88].

Cross-sectional imaging techniques, such as magnetic resonance enterography (MRE), ultrasonography (US), and computed tomography (CT), are used in conjunction with endoscopy to provide a comprehensive diagnostic evaluation and have shown favourable accuracy in diagnosing CD [83,84].

There are several radiological methods that help evaluate areas of the gastrointestinal tract that cannot be examined by endoscopic methods and provide additional information [85,87,89,90].

Abdominal X-rays are taken in the case of a suspicion of gastrointestinal obstruction, perforation, or acute intestinal distention.

The enterography and enteroclysis of the gastrointestinal tract, performed using CT or MRE, enable the assessment of the activity of the inflammatory process and the presence of complications (fistulas and abscesses) [85,88,91].

Ultrasound examination is a noninvasive method that allows for the evaluation of abdominal organs and inflammation of the intestinal wall.

Fistulography is an examination using contrast, allowing for the assessment of the course of the fistula [79,85,92].

All imaging studies are capable of detecting stenosis with a high degree of precision, with the selection of the best approach depending on availability, cost, the patient’s clinical condition (including comorbidities), and radiation problems [83,88,90].

### 3.3. Laboratory Testing

Currently, we do not have a sufficiently sensitive and specific marker of intestinal disease that would clearly indicate a specific disease or be the gold standard for assessing the severity of changes in this area. The beginning of the laboratory diagnostic process is usually very typical and similar to the diagnosis of other diseases. The tests presented below are not specific and are only characteristic of intestinal problems. However, they are absolutely the first diagnostic step [80,83,85,92].

1. Peripheral blood count. This is one of the most commonly performed diagnostic tests, giving a broad picture of what may be going on in the body; special attention is paid here to platelets and hemoglobin.
2. CRP (C-reactive protein) (a marker of inflammation). If there is inflammation in the body, an elevated determination of C-reactive protein will appear in the result. If there is inflammation in the intestines, the results of the determination of CRP will also be abnormal. In addition to this, you can perform OB tests (Passer reaction), which is also an indicator of inflammation but much less sensitive and specific.
3. Determination of electrolytes, magnesium, sodium, potassium, and chloride.
4. Liver transaminases (ALAT (alanine aminotransferase) and ASPAT (aspartate aminotransferase)). Liver tests show how the liver functions but also allow one to determine whether the cause of the unpleasant symptoms reported by the patient is only the intestines or also the liver.
5. Determination of iron levels.
6. Faecal occult blood. A test is recommended especially for people over 50 years of age; it is also indicated in any suspected gastrointestinal disorders; it is recommended to perform this determination first.
7. Determination of albumin level.
8. Determination of bilirubin level.
9. Determination of total protein levels [80,83,85,92,93].

The basic parameters of the laboratory tests are presented in Table 3.

### 3.4. Advanced Markers of Intestinal Inflammation

Advanced markers of intestinal inflammation:

– Antibodies against pancreatic exocrine cells: anti-rPAg1 (CUZD1) and anti-rPAg2 (GP2);
– Antibodies against oligomannose elements of the yeast cell wall-p/ASCA (anti-Saccharomyces cerevisiae antibodies) determined in IgA and IgG classes [85,94];
– Antibodies against cytoplasmic granules of neutrophils-p/ANCA and ASCA antibodies (determined as IgA and IgG) [79,94].

To properly perform the diagnostic process, additional tests must be performed, such as occult faecal blood tests; intestinal flora composition tests; residual digestion level tests; consistency and pH of stool tests; digestive parameter tests, such as for pancreatic elastase and bile acids; and celiac disease tests [83,85].

A recently published study showed that a higher serum erythrocyte sedimentation rate and platelet count, but not CRP, were associated with strictures in CD patients [95].

### 3.5. Biomarkers in the Stool

Unlike serum markers, the levels of which can be caused by lesions not only in the gastrointestinal tract, markers determined in the stool will originate from the inflamed intestinal mucosa or result from the interaction of the intestinal barrier with the altered intestinal microflora. These markers are particularly useful in the diagnosis of Crohn’s disease, where inflammatory changes in the gastrointestinal tract are often segmental, and it is sometimes difficult to assess the entire intestine on endoscopic examination [80,83,96].

#### 3.5.1. Calprotectin

Calprotectin is one of the most popular and frequently determined markers. It is found in neutrophils and is resistant to bacterial degradation. Since the concentration of calprotectin in the stool is directly proportional to the severity of neutrophil infiltration in the intestinal mucosa, it is an extremely sensitive indicator of the severity of inflammation (the intestinal barrier is unsealed, calprotectin-secreting leukocytes are infiltrated from the circulation, and the calprotectin concentration increases significantly). Calprotectin is a protein that binds calcium and zinc but is not a specific marker. This test does not differentiate the cause of inflammation; it answers the question of whether something is going on in the gut, not specifically “what is going on in the gut”. In healthy people, it is detected only in trace amounts. The concentration of calprotectin itself usually correlates well with the severity of inflammatory changes, so this determination is made as one of the first. If the determination of the result of the calprotectin is within the range of reference values, the implementation of invasive diagnostic methods, such as colonoscopy, for example, is generally waived [83,85,93].

In addition to being one of the first tests in the diagnosis of intestinal diseases, the calprotectin assay is also used to monitor the treatment of Crohn’s disease. An increase in calprotectin levels is also observed in other specific diseases, such as cancer, gastrointestinal polyps, infections, small intestinal microflora overgrowth (SIBO), liver cirrhosis, acute pancreatitis, and also in people who use frequent and long-term nonsteroidal anti-inflammatory drugs (e.g., ibuprofen or aspirin).

The determination of calprotectin also allows an initial differentiation of IBD from irritable bowel syndrome. Calprotectin can be determined in both faeces and blood. However, for the diagnosis of intestinal diseases, the emphasis is definitely on determining faecal calprotectin. Concentrations below 50 μg/g indicate the absence of an inflammatory process in the intestines, while concentrations above 150 μg/g suggest an ongoing inflammatory process within the intestines. Intermediate results are inconclusive and require a repeat control determination [83,85,92]. A recent article discusses the use of testing faecal calprotectin levels as a potential biomarker of stenosis in Crohn’s disease, demonstrating that it can serve as a complementary tool to ultrasound findings to confirm the therapeutic response [97].

#### 3.5.2. Alpha-1-Antitrypsin

Alpha-1-antitrypsin is a protease inhibitor produced primarily in the liver and, to a lesser extent, in the intestinal mucosa. Its increase is assumed to be associated with an increase in mucosal permeability. The determination of alpha-1-antitrypsin is performed in the stool. The elevated results of these determinations in relation to intestinal protein loss (also known as exudative enteropathy or enteropathy with protein loss) and with inflammatory lesions of the intestinal mucosa make it possible to detect even latent inflammatory reactions that do not yet produce specific clinical symptoms. However, it should be noted that allergic reactions to certain foods or toxins secreted by bacteria residing in the gastrointestinal tract can also result in increased permeability and, thus, increased alpha-1-antitrypsin in the stool. The alpha-1-antitrypsin concentration test faecal is a typical screening test for the diagnosis of chronic protein loss. The concentration of alpha-1-antitrypsin is high in meconium (stool of newborns), so the test in the first days of life is unreliable. Furthermore, in the case of alpha-1-antitrypsin, it is necessary to remember to distinguish between faecal and blood determinations [83,85,92].

#### 3.5.3. Lactoferrin

Lactoferrin is a glycoprotein that binds iron. It is found in large amounts in activated neutrophils that are involved in the inflammatory response. Lactoferrin is released from damaged intestinal tissue where inflammation occurs and also as an antimicrobial defence. Like calprotectin, discussed above, lactoferrin is not a specific marker of inflammatory bowel disease. However, it is very useful in the differential diagnosis of active and inactive forms of Crohn’s disease.

A negative result indicates the absence of inflammation, while a positive result indicates inflammatory activity but does not show the site of inflammation. Therefore, if a positive result is obtained, additional diagnostics should be performed [83,85].

### 3.6. Microbiological Tests

Bacterial and viral infections of the gastrointestinal tract are also risk factors for Crohn’s disease. Therefore, when CD is suspected, it is recommended to confirm or exclude other gastrointestinal diseases, including infections with Salmonella, Shigella, an enteropathogenic strain of *Escherichia coli (EPEC*)*, Clostridium difficile, Campylobacter jejuni, Yersinia enterocolitica, Helicobacter pylori* or *cytomegalovirus (CMV*) [80,94]. Before the introduction of immunosuppressive drugs, tests for hepatitis B and C infections (HBV and HCV, respectively), human immunodeficiency virus (HIV), and tuberculosis should be performed [85,92].

**Figure 2:**
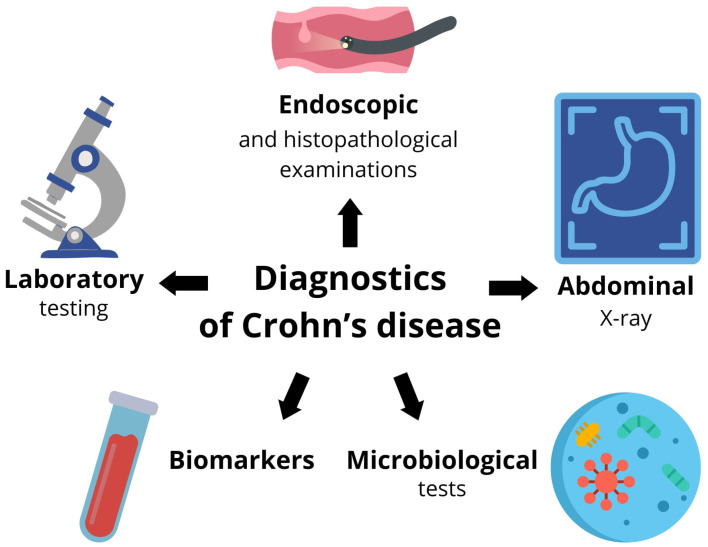
Selected diagnostic methods for Crohn’s disease.

A summary of the diagnosis of Crohn’s disease is shown in Figure 2.

## 4. Molecular Studies

One of the most common and serious complications of CD is intestinal fibrosis, which occurs in more than one-third of patients and leads to obstruction due to emerging strictures [98,99]. Complications caused by fibrosis are associated with high morbidity and mortality and lead to a significant number of hospitalisations and surgical procedures, generating high healthcare costs [85]. Chronic inflammation is an important initiating factor in fibrosis [100,101]. Studies have confirmed a strong association between inflammation and fibrosis in patients with CD [101,102]. Fibrosis in CD should be considered a result of the response (over) of the intestinal wall to the presence of inflammation in the deep structures of the intestinal wall [103].

Causes increased morbidity and mortality, prolonged hospitalisation, and the need for surgery [104]. Intestinal fibrosis has been found to develop despite the removal of the inflammatory stimulus and elimination of inflammation. This means that early intervention alleviates but does not eliminate subsequent fibrosis, suggesting that once initiated, fibrosis is self-perpetuating, so very early preventive interventions may have the greatest impact on the development or inhibition of fibrotic disease [100]. A key feature of fibrosis is the passage and accumulation of activated fibroblasts in the intestinal mucosa, which produce the collagen found in fibrotic tissue [105].

Fibrogenesis is a pathophysiological process during which the body responds to all kinds of damage caused by harmful factors, such as physical, chemical, and mechanical injuries, infections, and autoimmune diseases. The wound healing process requires the intervention of a large number of molecular and cellular components, which leads to the deposition of connective tissue in the ECM as a response to damage and results in tissue regeneration and repair. Fibrosis is precisely characterised by the excessive accumulation of ECM proteins in damaged areas [101,102]. The fibrosis process depends on the balance between ECM synthesis and degradation. Fibrosis occurs when synthesis exceeds degradation, while the intestine begins to heal without fibrosis when this balance is reversed [106]. Although inflammation is necessary for the development of fibrosis, controlling intestinal inflammation alone cannot stop the development of fibrosis, suggesting that chronic inflammation is not the only cause of fibrogenesis [107]. Several mechanisms independent of inflammation are involved in the pathogenesis of fibrosis, including the intestinal microbiota, creeping fat, the interaction of the ECM, and metabolic reprogramming [107,108,109,110,111,112,113].

The main cells involved in fibrogenesis are mesenchymal cells, characterised by high motility and versatility, which are also important in collagen production [85,94,98]. Inflammation has been found to be the strongest activator of mesenchymal cells, initiating fibrogenesis, both in the early stages of CD and during the course of the disease, eventually leading to fibrotic scarring that can permeate the entire tissue architecture [114]. When the stimulus for fibrogenesis is persistent or recurrent, or even abnormal or excessive, as in Crohn’s disease, persistent tissue damage and subsequent healing processes lead to scar tissue [84]. Young fibroblasts begin to accumulate and gradually progress to fibrosis [112]. This process can proceed in an uncontrolled manner [98,115]. This, in turn, can result in tissue fibrosis and scarring, with irreversible anatomical and/or functional changes that ultimately cause intestinal obstruction. In addition, slow blood flow due to damage to highly branched vessels can often induce drug resistance [101]. The mechanisms underlying intestinal fibrosis are only partially known. Pathologically, intestinal fibrosis in Crohn’s disease is distinguished by the accumulation of ECM and the expansion of mesenchymal cells, affecting all layers of the intestinal wall along its length [84].

Currently, there is no clinical therapy to prevent or reverse fibrosis; anti-inflammatory drugs do not prevent or cure fibrosis in IBD, even if they relieve inflammation, making surgical treatment the main treatment for fibrosis and intestinal stenosis [98,100,101]. As a key complication of IBD, intestinal fibrosis is common and a great challenge in the treatment process. At initial diagnosis, at least 10% of CD patients have a fibrotic stenosis phenotype. However, up to 50% of patients with CD eventually develop stricture or penetrating complications, and 70% of patients require surgery within 10 years of diagnosis [85,100,103,107,116]. In addition, stenosis recurs at anastomotic sites in up to 50% of patients, requiring multiple subsequent surgeries. Fibrosis is generally considered permanent and irreversible, and very few treatments for fibrosis are available, most with limited efficacy [84,103]. In recent decades, despite the availability and efficacy of biological therapies for IBD, the incidence of intestinal stenosis has not decreased significantly [117]. This means that pure anti-inflammatory treatment does not necessarily alleviate the associated fibrosis. Fibrotic complications that lead to stenosis, intestinal obstruction, and the need for surgical intervention represent one of the biggest unresolved clinical problems in IBD [116,118]. The treatment of intestinal tissue damage is a major therapeutic challenge in CD patients [116]. One of the key issues in intestinal fibrosis research is the search for biomarkers to detect this process in patients as soon as possible [119]. This discovery would help significantly in the application of personalised and timely treatment [116]. In addition to colonoscopy and endoscopy, experiments are being conducted at the molecular level, investigating, among other things, the role of miRNAs in fibrosis [100].

MicroRNAs (miRNAs) are small noncoding ribonucleic acid (RNA) sequences of approximately 24–30 nucleotides long, forming RNA–protein complexes that interfere with mRNA and, in most cases, cause translation inhibition [120,121]. The role of miRNAs in intestinal fibrosis in CD is relatively poorly understood; at least two families of miRNAs, miRNA-29 and miRNA-200, are believed to be involved. MiRNA-29a, -29b, and -29c have been found to decrease in the CD constricted mucosa. MiRNA-29b plays a role in modulating in vitro expression of collagen I and III. On the contrary, the miRNA 200 family is thought to play a protective role against the development of the epithelial–mesenchymal transition (EMT) and may inhibit the development of fibrosis [98,122,123]. Fibrosis is a constantly evolving process in which epithelial cells acquire a migratory function that is sustained precisely by EMT [124]. Other studies have shown that miRNA-16 also prevents intestinal cell migration and, thus, fibrosis [125].

A study by Lewis et al. identified miRNA-19-3p as a potential marker of constriction in CD. Patients with CD stenosis had reduced serum levels of miRNA-19a-3p and miRNA-19b-3p compared to controls. The association between miRNA-19-3p and CD stenosis appeared to be independent of clinical factors such as disease duration, disease activity, location, gender, or age. A 4-year follow-up of patients confirmed this hypothesis [126].

Similarly, miRNA-320 can promote epithelial repair, and its deficiency is believed to be a marker of an increased inflammatory response [120]. The usefulness of miRNAs in the diagnosis of IBD was also confirmed in another prospective study involving 77 patients with IBD. The authors found that miRNA-320a blood levels were strongly correlated with CD exacerbation and reflected endoscopic and clinical disease activity [127].

A study was conducted in Slovenia in 2019 using samples from 30 CD patients who underwent bowel resection. The control group consisted of healthy intestinal samples (colon and ileum) from 10 patients who underwent resection for colon cancer. The selected miRNAs, which have been shown to be involved in inflammation and fibrosis in various organs, showed a statistically significant deregulation of miRNA-29c and miRNA-155 in samples from fibrotic areas and a statistically significant deregulation of miRNA-150 and miRNA-155 in samples from inflamed areas in CD compared to normal mucosa [103].

A growing body of the literature suggests that most microRNAs, including miRNA-29c, miRNA-150, and miRNA-155, contribute to a broad spectrum of disease processes. Furthermore, numerous publications have shown that panels of microRNAs, not just a single microRNA, are needed to detect the appropriate signature of a disease or pathogenetic process [128]. Additional studies are warranted to expand the knowledge about the involvement of microRNAs in CD.

It is speculated that miRNAs may, in the future, become biomarkers for an early noninvasive diagnosis in the treatment of intestinal fibrosis [129]. It is stated that the research performed so far still warrants confirmation in more robust studies due to the small sample sizes, the lack of control of the heterogeneity of the patients, and the absence of a standard protocol to assess miRNAs [114].

A study was conducted on the transcriptional regulator ZNF281, which induces the epithelial–mesenchymal transition (EMT). The purpose of this study was to investigate in vivo and in vitro the role of ZNF281 in intestinal fibrogenesis. Intestinal fibrosis was studied in vivo in mice with chronic colitis. The involvement of ZNF281 in intestinal fibrosis was also studied in vitro in a human colonic fibroblast cell line activated with the profibrotic cytokine TGFβ1. The results showed a significant increase in ZNF281 levels in mouse colonic fibrosis in vivo, as well as in human colonic fibroblasts activated by TGF-1 in vitro that are, in turn, activated by TGFβ1 [130].

Inflammation causes TGFβ release and consequent activation of fibroblasts, which differentiate into myofibroblasts, a cell type with a phenotype intermediate between smooth muscle cells and fibroblasts. Myofibroblasts have both contractile and migratory capacities and are responsible for the synthesis of ECM proteins [130].

Given that the hallmark of CD strictures is excessive ECM secretion and an increase in the number of mesenchymal cells in various areas of the intestinal wall, ECM-producing cells, namely fibroblasts and myofibroblasts, are crucial in fibrosis [114].

In the study cited, ZNF281 was shown to be a key regulator of colonic fibroblast activation and myofibroblast differentiation under fibrotic stimuli through transcriptional control of ECM composition, remodelling, and cell shrinkage, highlighting a novel role in the onset and progression of intestinal fibrosis.

ZNF proteins are one of the most abundant groups of proteins that play a key role in regulating important cellular processes. Changes in ZNF are involved in the development of many human diseases and cancer progression, including colorectal cancer; in particular, it is ZNF281. ZNF281 levels have been shown to increase significantly in colitis in patients with IBD, and preliminary data suggest their potential impact on intestinal fibrogenesis.

The cited study shows, for the first time, that the transcription factor ZNF281 is involved in TGFβ1-induced intestinal fibrosis. Although the mechanism is still unclear, the authors believe that the inhibition of ZNF281 may provide a therapeutic strategy to alleviate fibrosis by altering fibroblast activation and collagen deposition. ZNF281 was found to act as a transcriptional activator of profibrotic genes involved in myofibroblast differentiation, and TGFβ1 was found to regulate the expression mediated by ZNF281 of fibrosis-related genes, mainly involved in cell differentiation and migration.

However, as the authors of the cited study point out, the results are still limited and nonexhaustive [130].

Another study observed that TGFβ1-induced collagen expression is reversed by the exogenous overexpression of miRNA29b. In addition, miRNA200 has been shown to partially protect intestinal epithelial cells from fibrogenesis and pseudo-inhibition of EMT [98].

Currently, genetic testing is not recommended in the routine diagnosis of CD, and laboratory diagnostics may be helpful in assessing the severity and complications of the disease [19,92].

## 5. Conclusions

Intestinal fibrosis is one of the most serious complications of Crohn’s disease. It occurs in more than a third of patients with the disease, is associated with increased morbidity and mortality, and surgery is often the only available therapeutic option. The mechanisms underlying intestinal fibrosis are only partially understood. Previous studies have demonstrated an important pathogenetic role for mesenchymal cells (especially myofibroblasts), cytokines (e.g., transforming growth factor β), growth factors, microRNAs, the intestinal microbiome, crawling fat, matrix stiffness, and mesenteric adipocytes. More research using various advanced techniques is needed to elucidate all the mechanisms involved in intestinal fibrosis so that personalised therapies can be developed and implemented.

The ability to identify markers of intestinal fibrosis would help develop new diagnostic and therapeutic methods to detect the early stages of fibrosis, reduce symptoms, and assess patients’ risk of acquiring fibrosis. Due to conflicting research results and the availability of low-quality scientific data, there is currently no reliable biomarker that can be used to predict fibrosis in routine clinical practice.

Despite the uncertainty of future advances in the field, it is not doubtful that the evaluation and diagnosis of fibrosis in IBD require a multidisciplinary approach involving gastroenterologists, radiologists, pathologists, surgeons, nurses, and laboratory diagnosticians. The discovery of therapies that aim to reverse or prevent intestinal fibrosis will be a major breakthrough in medicine.

## Figures and Tables

**Table 1:** Degree of disease activity according to the European Crohn’s and Colitis Organisation (ECCO) [13].

Disease Activity	Equivalent to a CDAI	Examples of Parameters That Are Evaluated
Mild	150–220	weight loss < 10%, no features of obstruction, absence of fever;
Moderate	220–450	weight loss > 10%, occasional vomiting;
Severe	>450	BMI < 18 kg/m^2^, intestinal obstruction, abscess

**Table 2:** Montreal classification of Crohn’s disease [15,16,17].

	Montreal Classification
Age at diagnosis	A1 below 16 years of age
	A2 between 17 and 40 years of age
	A3 above 40 years of age
Location	L1 ileal
	L2 colonic
	L3 ileocolonic
	L4 isolated upper disease ^1^
Behaviour	B1 nonstricturing, nonpenetrating
	B2 stricturing
	B3 Penetrating
	p perianal disease modifier ^2^

^1^ Add to L1–L3 when there is concurrent upper gastrointestinal disease. ^2^ Should be added to B1–B3 when there is concurrent perianal disease.

**Table 3:** Basic parameters of laboratory tests [83].

Parameters	Indicative Reference Values
Hemoglobin	F: 12.0–16.0 g/dL M: 13.0–18.0 g/dL
Red blood cells	F: 3.5 × 10^12^–5.2 × 10^12^/L M: 4.2 × 10^12^–5.4 × 10^12^/L
Hematocrit (ratio of erythrocyte volume to total blood volume)	F: 37–47% M: 40–49%
Leukocytes	4 × 10^9^–10 × 10^9^/L
Platelets	150 × 10^9^–450 × 10^9^/L
Reticulocytes	20 × 10^9^–100 × 10^9^/L
Average hemoglobin concentration in erythrocytes	32–36 g/dL
CRP (reactive protein C)	0.08–3.1 mg/L
Iron	60–180 μg/dL
Albumin	35–50 g/L
Total bilirubin	0.3–1.0 mg/dL
Total serum protein	60–80 g/L
